# The Effects of Being an Only Child, Family Cohesion, and Family Conflict on Behavioral Problems among Adolescents with Physically Ill Parents

**DOI:** 10.3390/ijerph120910910

**Published:** 2015-09-02

**Authors:** Guo-Yuan Sui, Jia-Na Wang, Guang-Cong Liu, Lie Wang

**Affiliations:** 1 School of Public Health, China Medical University, No.77 Puhe Road, Shenyang North New Area, Shenyang 110013, China; E-Mails: lgc0519@126.com (G.-Y.S.); jiana0818@163.com (J.-N.W.); 2 Shenyang Academy of Environmental Sciences, No.139 NantaStreet, Shenhe District, Shenyang 110000, China; E-Mail: lgc0519@sina.com

**Keywords:** only child, adolescent, family cohesion, family conflict, behavioral problems, physically ill parents

## Abstract

Background: This study aimed to examine the parental physical illness’ effect on behavioral problems among adolescents, and the effects of being an only child, family cohesion, and family conflict on behavioral problems among adolescents with physically ill parents in Liaoning province, China. Methods: This cross-sectional study was performed in 2009. A questionnaire including two dimensions of the Family Environment Scale (family cohesion and family conflict), self-reported Strength and Difficulties Questionnaire (SDQ), and demographic factors was distributed to the subjects. Results: Among the 5220 adolescents, 308 adolescents lived with physically ill parents. The adolescents with physically ill parents had more behavioral problems than adolescents with healthy parents. Among the girls who lived in families with physically ill parents, the SDQ score and the prevalence of SDQ syndromes were higher in the girls with siblings than the girls without siblings after adjusting for variables; the effect of family cohesion on SDQ was significant after adjusting for variables. Conclusion: Interventions targeting family cohesion may be effective to reduce behavioral problems of adolescents with physically ill parents.

## 1. Introduction

Living with physically ill parents for adolescents is frequency, as 5%–15% children and adolescents may live with a parent who suffers from physical illness (the US National Center for Health Statistics) [1]. It is challenging for adolescents to live with a physically ill parent. They need to take on additional family responsibilities during parental illness [2,3]. Families with physically ill parents may suffer the loss of financial resources [4]. Due to these factors, adolescents with physically ill parents may be at a high risk of behavioral problems [5,6,7]. Studies reported that adolescents with physically ill parents may have more internalizing problems (such as anxiety and depressed mood) and externalizing problems (such as aggressive behavior and delinquent behavior) compared to adolescents with healthy parents [5,6,7]. Adolescents who have behavioral problems are vulnerable to psychiatric disorders in their adulthood [8,9].Therefore, it is important to focus on behavioral problems among adolescents with physically ill parents. However, a substantial body of research in adolescents with physically ill parents was performed in Europe and America to date, and few studies were conducted in China. In this study, we hypothesized that Chinese adolescents with physically ill parents would have more behavioral problems.

Previous studies have demonstrated that being an only child was associated with behavioral problems. The Chinese government implemented the “One child policy” in early 1979. Therefore there are now a large proportion of adolescents without siblings in China. Some studies from Britain, Korea, and Netherlands have shown that children without siblings are overprotected and self-centered, which may have a negative effect on their psychological development [10,11,12]. In China, researchers have found that children without siblings might have more behavioral problems than children with siblings [13,14]. However, Liu *et al*., I found that compared to adolescents without siblings, adolescents with siblings had worse mental health in China based on resource dilution theory (family resources are divided by the number of children, and increasing number of children may lower quality of the output) [15].The quality of output means that children obtain resources such as economic resources and interpersonal resources from family. Compared to the children with siblings, the children without siblings obtain more economic and interpersonal resources (such as attention, time, and energy), which may be conductive to their well-being [15]. Moreover, Li *et al*., reported that there were no significant differences for behavioral problems between children with and without siblings [16]. Although there were many studies exploring the effects of being an only child on adolescents’ mental health, most studies were limited to general population, and few of them focused on adolescents with physically ill parents. Parental physical illness may lead to the loss of financial resources and less attention to adolescents from family members [4,17]. Loss of family resources may enhance the negative effect of sibship size on children’s development [18]. In addition, due to the implementation of “one child policy” in China, family with two or more children would suffer financial punishment and family with only one child can be encouraged, such as obtaining more financial aid for medical problems [19]. All above facts seem to deteriorate the mental health of children with siblings living in families with physically ill parents. Therefore, we hypothesized that Chinese children with siblings living in families with physically ill parents would have more behavioral problems.

Family cohesion is defined as “the degree of commitment and support family members provide for one another”; family conflict is defined as “the amount of openly expressed anger and conflict among family members” [20]. Kissane *et al*., reported that family cohesion was an important element of family coping [21]. Previous studies have suggested that high levels of support and low levels of conflict may help family members better cope with physical illness [22,23,24,25]. Parental physical illness may have a negative effect on adolescents’ behavioral problems [5,6,7]. Adolescents from high cohesive and low conflictive family may better psychologically adjust to physical illness [23,24,25]. To date, most studies exploring the effects of family cohesion and conflict on adolescents’ behavioral problems were conducted in western societies. A study from 68 countries demonstrated that China was more collectivistic than western countries [26]. Collectivism focuses on community and emphasizes the importance of cohesion [27]. In China, family is considered a community. Family members are more likely to support each other and less likely to express their anger [28]. Greenberger *et al*., found that the relationship between the quality of family relationship and depressive symptoms was stronger among Chinese adolescents than among US adolescents [29]. Therefore, we hypothesized that family cohesion and family conflict would have effects on behavioral problems of adolescents with physically ill parents in China.

There are three aims in this study. First, we examined whether adolescents with physically ill parents had more behavioral problems compared to adolescents with healthy parents in China. Second, we assessed the effects of being an only child on adolescents’ behavioral problems in the families with physically ill parents. Third, we explored the effects of family cohesion and family conflict on adolescents’ behavioral problems in families with physically ill parents.

## 2. Method 

### 2.1. Sample

A survey was conducted in Liaoning province, China in 2009. According to population size, Liaoning province comprises three metropolitan cities (≥1,000,000), seven medium-size cities (500,000–1,000,000) and four small cities (200,000–500,000).Three cities (one metropolitan, one medium-size and one small city) were randomly selected in 2009.Three urban areas from each city and two rural areas from the large and medium-size cities were randomly selected. Rural areas in small city with few public schools were not included. Six public schools (two primary schools (grades 5–6) and four middle schools (grades 7–12)) were randomly selected from each area by age range (11–18 years), and two or three classes were randomly selected from each grade. We randomly selected 30 students (15 boys and 15girls) in each selected class using a pre-prepared list of random numbers. Parental illness was reported by the parents. Parents with mental health illness were excluded. Among the 5220 adolescents (boys: 2277 (43.6%); mean age: (13.74 ± 2.10) years; age range: (11–18 years), 308 adolescents lived with physically ill parents. Among these 308 adolescents, 172 had fathers with physical illness; 175 had mothers with physical illness; 39 had both parents with physical illness. The details of parental physical illness were presented in Table 1. Four thousand one hundred and three (78.6%) were only children; 4147 (79.4%) lived in urban areas; 4777 (91.5%) lived with both biological parents; as to family income, the proportions of “<500 RMB”, “500–1000 RMB”, “1001–1500 RMB”, “1501–2500 RMB”, “>2500 RMB” were 7.9%, 19.9%, 23.3%, 22.7% and 26.1% respectively; as to father’s educational level, the proportions of junior high school level, senior high school level, and college level or higher were 41.2%, 40.9%, and 17.9% respectively; for mother’s educational level, the proportions of junior high school level, senior high school level, and college level or higher were 43.1%, 40.8% and 16.1% respectively. The average age of the fathers was (40.50 ± 4.10) years; the average age of the mothers was (39.02 ± 3.84) years. All the participants were informed about the purpose of the survey before their participation. The procedures were approved by the Ethics Committee on Human Experimentation of China Medical University. Written informed consent was obtained from all participating adolescents and their parents (CMU62083004). 

**Table 1:** Detailed information of parental physical illness.

Disease	Father (172)*n* (%)	Mother (175)*n* (%)
Cancer	4 (2.3)	5 (2.9)
Rheumatism	10 (5.8)	18 (10.3)
Stomach/Liver/Gallbladder/Pancreas/Kidney disease	21 (12.2)	13 (7.4)
Lung/Weasand disease	5 (2.9)	5 (2.9)
Heart disease	25 (14.5)	57 (32.6)
Cerebropathy	13 (7.6)	4 (2.3)
Hypertension	32 (18.6)	20 (11.4)
Diabetes	31 (18.0)	9 (5.1)
Cervical/lumbar spondylosis	22 (12.8)	30 (17.2)
Hyperthyroidism	-	4 (2.3)
Anemia	-	6 (3.4)
Two or more diseases	9 (5.2)	4 (2.3)

### 2.2. Instruments

#### 2.2.1. The Self-Report Version of the Strengths and Difficulties Questionnaire (SDQ)

Behavioral problems were measured with the self-reported SDQ. SDQ contains 25 items and can be responded to on a three-point Likert scale, with response categories ranging from “no” (0 points) to “somewhat” (2 points) [30]. The self-reported SDQ includes five factors: emotional symptoms (5 items, e.g., “I get a lot of headaches, stomach-aches or sickness”; “I worry a lot”), conduct problems(5 items, e.g., “I get very angry and often lose my temper”; “I usually do as I am told”), hyperactivity/inattention (5 items, e.g., “I am restless, I cannot stay still for long”; “I am constantly fidgeting or squirming”), peer problems (5 items, e.g., “I am usually on my own. I generally play alone or keep to myself”; “I have one good friend or more”), and prosocial behavior (5 items, e.g., “I try to be nice to other people. I care about their feelings”; “I usually share with others (food, games, pens, *etc.*)”) [31]. All factors except the prosocial behavior are summed to assess behavioral problems [31]. For behavioral problems, we applied the cut-off score of 18 to define the “behavioral problems” group [32]. The Cronbach’s alphas for the whole questionnaire were 0.69 and 0.71 in adolescent with and without physically ill parents, respectively.

#### 2.2.2. Family Environment Scale (FES)

Family cohesion and family conflict were measured with the FES, which was filled in by adolescents. The FES is a 90-item true-false measure which forms 10 factors [33]. In this study, we focused on two factors, cohesion and conflict. Each of these factors contains nine items. The sample items of family cohesion included “Household members really help and support one another”, “We put a lot of energy into what we do at home” and so on. The sample items of family conflict included “We fight a lot in our household”, “Household members often criticize each other” and so on.

The Cronbach’s alphas for family cohesion were 0.74 and 0.78 in adolescent with and without physically ill parents, respectively; The Cronbach’s alpha for family conflict were 0.63 and 0.72 in adolescent with and without physically ill parents, respectively.

#### 2.2.3.Demographic Factors

Demographic factors included adolescent’s age, adolescent’s gender, being an only child, living area(urban/rural), family structure, family income (<500 RMB/500–1000 RMB/1001–1500 RMB/1501–2500 RMB/>2500 RMB), father’s/mother’s age and father’s/mother’s educational levels (junior high school level/senior high school level/college level or higher). “Family structure” was divided into two groups of “intact family” (living with both biological parents) and “non-intact family” (living with single parent, step-parent, or foster parent) [34]. Adolescent’s age, adolescent’s gender, being an only child, family structure and living area were completed by adolescents. Father’s/mother’s educational levels and family income were completed by parents. Previous studies reported that adolescent age, adolescent gender, living area, family structure, family income, father’s/mother’s age, father’s/mother’s educational levels were associated with behavioral problems [34,35,36,37]. Therefore, in this study, we adjusted for these variables.

### 2.3.Statistical Analysis

Chi-square analyses for dichotomous variables, and independent-sample *t*-tests and one-way ANOVA analyses for continuous variables were used to examine differences of demographic variables and behavioral problems between adolescents with and without physically ill parents, and among the adolescents with physically ill father, physically ill mother, and both parents with physical illness. The distributions of behavioral problems in categorical variables were examined by the independent-sample *t*-tests, one-way ANOVA analyses and Chi-square analyses. Correlations among behavioral problems and all continuous variables were examined by Pearson correlation. In the total sample, the effects of parental physical illness and being an only child on adolescents’ behavioral problems were examined using analyses of covariance; we then performed logistic regression analyses to examine the effects of parental physical illness and being an only child on adolescents’ behavioral problems. The interaction terms of only child with parental physical illness on behavioral problems were examined by logistic regression analyses and analyses of covariance. In the families with and without physically ill parents, after adjusting for adolescent’s age, adolescent’s gender, family structure, living area, father’s/mother’s educational levels, father’s/mother’s age and family income, the effects of being an only child on adolescents’ behavioral problems was examined using analyses of covariance; we then performed logistic regression analyses to examine the effects of being an only child on adolescents’ behavioral problems. The interaction terms of family cohesion and family conflict with parental physical illness on behavioral problems were examined by multiple linear regression analyses. In the families with and without physically ill parents, we used multiple linear regression analyses to examine the effects of family cohesion and family conflict on adolescents’ behavioral problems.

The analyses were performed with SPSS13.0, with two-tailed probability value of <0.05 considered to be statistically significant.

## 3. Results

There were no differences in demographic characteristics (including adolescent’s gender, adolescent’s age, being an only child, living area, family structures, family income, father’s education levels, father’s age, and mother’s age) and behavioral problems except mother’s education levels among adolescents with physically ill father, physically ill mother and both parents with physical illness (*p >* 0.05). There were no differences between the adolescents with and without physically ill parents in terms of adolescent’s gender, adolescent’s age, being an only child, and living area. The distributions of family income, family structures, and father’s/mother’s education level were significantly different between the adolescents with and without physically ill parents (*p >* 0.05). In the family with physically ill parents, parents reported older age, lower family income and education levels; adolescents reported that a smaller proportion of them lived with both biological parents compared to children with healthy parents (*p <* 0.05).

Based on the results from Chi-square analyses, independent-sample *t*-tests and one-way ANOVA analyses, being an only child was associated with behavioral problems among adolescents with physically ill parents; living area and father’s/mother’s education were associated with behavioral problems among the adolescents without physically ill parents; gender was associated with behavioral problems among the adolescents without physically ill parents; family structure was associated with behavioral problems among the girls without physically ill parents; adolescents with physically ill parents had more behavioral problems (*p <* 0.05). Based on independent-sample *t*-tests and one-way ANOVA analyses, we also found that family structure was correlated with behavioral problems among the boys without physically ill parents; family income was correlated with behavioral problems among the girls without physically ill parents; being an only child was related to behavioral problems among the girls without physically ill parents (*p <* 0.05).

Adolescent’s age was associated with hyperactivity/inattention among the boys with physically ill parents; adolescent’s age was associated with behavioral problems and emotional symptoms among the girls with physically ill parents; adolescent’s age, father’s age and mother’s age were related to behavioral problems, emotional symptoms, conduct problems, and hyperactivity/inattention among the adolescents without physically ill parents; father’s age was also related to peer problems among the girls without physically ill parents (*p <*0.05). Family cohesion was associated with behavioral problems, emotional symptoms, conduct problems, and hyperactivity/inattention among the adolescents with physically ill parents; family cohesion was also related to prosocial behavior and peer problems among the boys with physically ill parents and the girls with physically ill parents, respectively; family conflict was associated with behavioral problems, emotional symptoms, conduct problems, and hyperactivity/inattention among adolescents with physically ill parents; family conflict was also related to peer problems and prosocial behavior among boys with physically ill parents; family cohesion and conflict were associated with total scale and each sub-scale of behavioral problems among the adolescents without physically ill parents (*p <* 0.05).(shown in Supplementary Materials and Research Data).

The effect of parental physical illness on behavioral problems in the total study population is presented in Table 2. In the total study population, the effect of parental physical illness on behavioral problems was significant after adjusting for variables. Adolescents with physically ill parents had more behavioral problems compared to the adolescents without physically ill parents.

**Table 2:** The effect of parental physical illness on behavioral problems in the total sample.

Behavioral Problems	Adolescents without Parental Physical Illness	Adolescents with Parental Physical Illness	Adjusted *F* ^a^	Adjusted ^b^ OR (95%CI)
Boys (*n =* 2277)				
Mean (SD)	11.22 (5.86)	12.95 (6.47)	6.82 *	-
%	14.2%	21.6%	-	1.60 (1.02–2.53)
Girls (*n =* 2943)				
Mean (SD)	10.55 (5.59)	12.74 (5.32)	11.69 *	-
%	11.2%	20.7%	-	1.68 (1.09–2.60)
Total (*n =* 5220)				
Mean (SD)	10.84 (5.72)	12.83 (5.86)	18.51 *	-
%	12.5%	21.1%	-	1.64 (1.19–2.25)

* *p <* 0.05; **^a^**: adjusting for adolescent’s age, adolescent’s gender, only child, family structure, living area, father’s/mother’s educational level, father’s/mother’s age and family income in analyses of covariance among the total samples; all these variables excluding adolescent gender were controlled in analyses of covariance among boys and girls; **^b^**: adjusting for adolescent’s age, adolescent’s gender, only child, family structure, living area, father’s/mother’s educational level, father’s/mother’s age and family income in logistic regression analysis among the total samples; all these variables excluding adolescent gender were controlled in logistic regression analysis among boys and girls.

The interaction terms of being an only child with parental physical illness on behavioral problems were significant among the girls after adjusting for adolescent’s age, family structure, living area, father’s/mother’s educational levels, father’s/mother’s age and family income (F=4.06, *p <* 0.05, OR = 2.87, 95%CI: 1.15 to 7.12). But it wasn’t significant among boys (boys: F = 1.34, *p =* 0.25, OR = 1.03, 95%CI: 0.36 to 2.97). Compared to the girls without physically ill parents, being an only child had a stronger effect on behavioral problems among the girls with physically ill parents.

The effect of being an only child on behavioral problems in the total study population and the adolescents with and without physically ill parents is presented in Table 3. In the total study population and the adolescents without physically ill parents, the effect of being an only child on behavioral problems was not significant after adjusting for adolescent’s age, family structure, living area, father’s/mother’s educational levels, father’s/mother’s age and family income. Among girls with physically ill parents, the effect of only child on behavioral problems was significant after adjusting for adolescent’s age, family structure, living area, father’s/mother’s educational levels, father’s/mother’s age and family income (*p <*0.05). Compared to girls without siblings, girls with siblings had more behavioral problems.

**Table 3:** The effect of being an only child on behavioral problems in the total sample and adolescents with and without physically ill parents.

Behavioral Probelms	Only Child	Adolescents with Siblings	Adjusted *F* ^c^	Adjusted ^d^ OR (95%CI)
Total samples **^a^** (*n =* 5220)				
Boys (*n =* 2277)				
Mean (SD)	11.26 (5.93)	11.64 (5.84)	0.19	-
%	14.4%	15.7%	-	1.02 (0.73–1.44)
Girls (*n =* 2943)				
Mean (SD)	10.50 (5.46)	11.24 (6.01)	1.58	-
%	11.0%	14.1%	-	1.13 (0.84–1.52)
Total (*n =* 5220)				
Mean (SD)	10.84 (5.69)	11.38 (5.95)	1.68	-
%	12.5%	14.7%	-	1.08 (0.87–1.35)
Adolescents with physically ill parents **^b^** (*n =* 308)				
Boys (*n =* 139)				
Mean (SD)	12.67 (6.57)	13.94 (6.13)	2.40	-
%	21.3%	22.6%	-	2.11 (0.61–7.36)
Girls (*n =* 169)				
Mean (SD)	11.86 (4.89)	14.96 (5.76)	8.73 *	-
%	14.0%	37.5%	-	4.70 (1.69–13.02)
Total (*n =* 308)				
Mean (SD)	12.24 (5.74)	14.56 (5.90)	9.71 *	-
%	17.5%	31.6%	-	3.04 (1.42–6.50)
Adolescents without physically ill Parents **^e^** (*n =* 4912)				
Boys (*n =* 2138)				
Mean (SD)	11.17 (5.88)	11.45 (5.78)	0.01	-
%	14.0%	15.1%	-	0.97 (0.68–1.39)
Girls (*n =* 2774)				
Mean (SD)	10.42 (5.48)	10.96 (5.94)	0.36	-
%	10.8%	12.4%	-	0.98 (0.72–1.35)
Total (*n =* 4912)				
Mean (SD)	10.76 (5.67)	11.14 (5.88)	0.26	-
%	12.2%	13.4%	-	0.97 (0.77–1.23)

* *p <* 0.05; **^a^**: In the total samples, adjusting for adolescent’s age, adolescent’s gender, family structure, living area, father’s/mother’s educational level, father’s/mother’s age, parental physical illness and family income; all these variables excluding adolescent gender were controlled among boys and girls; **^b^** and **^e^**: Among adolescents with and without physically ill parents, adjusting for adolescent’s age, adolescent’s gender, family structure, living area, father’s/mother’s educational level, father’s/mother’s age and family income; all these variables excluding adolescent gender were controlled among boys and girls; **^c^**: analyses of covariance; **^d^**: logistic regression analyses.

The interaction terms of family cohesion and family conflict with parental physical illness on behavioral problems weren’t significant among boys and girls after adjusting for adolescent’s age, family structure, being an only child, living area, father’s/mother’s educational levels, father’s/mother’s age and family income (boys: family cohesion × parental physical illness, beta = −0.08, *p =* 0.82. family conflict × parental physical illness, beta = −0.23, *p =* 0.48; girls: family cohesion × parental physical illness, beta = 0.25, *p =* 0.28. family conflict × parental physical illness, beta = −0.25, *p =* 0.26). There was no difference in the effects of family cohesion and conflict on behavioral problems between the adolescents with and without physically ill parents. 

In the families with physically ill parents, after adjusting for adolescent’s age, family structure, living area, father’s/mother’s educational level, father’s/mother’s age and family income, the effect of family cohesion on behavioral problems was significant among boys and girls (boys: beta = −1.01, 95%CI: −1.84–0.18, standardized beta (SE) = −0.31 (0.42); girls: beta = −0.58, 95%CI: −1–0.16, standardized beta (SE) = −0.25 (0.21)). Family cohesion and family conflict explained 12% and 9% of the variance in the criterion variable in boys and girls, respectively. In the families without physically ill parents, after adjusting for adolescent’s age, family structure, living area, father’s/mother’s educational level, father’s/mother’s age and family income, the effects of family cohesion and family conflict on behavioral problems were significant among boys and girls (family cohesion: (boys: beta = −0.91, 95%CI: −1.07–0.75, standardized beta (SE) = −0.27 (0.08); girls: beta = −0.86, 95%CI: −1–0.72, standardized beta (SE) = −0.26 (0.07)); family conflict: (boys: beta = 0.63, 95%CI: 0.48–0.79, standardized beta (SE) = 0.19 (0.08); girls: beta = 0.57, 95%CI: 0.45–0.69, standardized beta (SE) = 0.19 (0.06))), accounting for 16% of the variance in the criterion variable in both boys and girls. Among adolescents with physically ill parents, adolescents from high cohesive families had low behavioral problems.

## 4. Discussion

In this study, we found adolescents with physically ill parents had more behavioral problems than adolescents with healthy parents in China. These results were consistent with previous studies that reported mental health problems in adolescents whose parents were affected by mixed physical illness and particular physical illness [5,6,7]. Adolescents living with physically ill parents may need to take more responsibilities, such as taking care of family members, and have less leisure time to play with friends, which may deteriorate their mental health [2,3]. In addition, parental physical illness may give rise to depletion of financial resources [29]. Compared to western countries, investment in health care in China is less, which may aggravate the financial burden [38,39]. Therefore, Chinese adolescents with physically ill parents may have more behavioral problems.

In families with physically ill parents, the girls with siblings were found to have more behavioral problems than the girls without siblings. However, we didn’t find that the effect of only child on adolescents’ behavioral problems was significant in the whole sample and boys with physically ill parents. Compared to the girls without physically ill parents, the effect of only child on behavioral problems was stronger among the girls with physically ill parents. Our results were inconsistent with Visser *et al*., which showed that children with no or few siblings were more vulnerable to behavioral problems than children with more siblings in the families with parents affected by cancer [40]. Multiple factors may contribute to the results. Families with physically ill parents may have to suffer the potential depletion of financial resources [4]. In families with ill parents, family members’ attention to adolescents may be less than that in families with healthy parents [17]. According to resource dilution theory, more economic and family interpersonal resources (such as attention, time, and energy) may help only children better adjust to stress and challenges [15].Additionally, girls tend to rely on their family as a source of emotional support [41]. Moreover, as part of traditional Chinese culture, gender discrimination against girls is still prevalent so that boys may get more attention compared to their sisters [19]. Therefore, in families with physically ill parents, the girls with siblings are more susceptible to behavioral problems. This increased risk may also exist in other Asian developing countries that share similar cultures. And due to the implementation of the “one child policy” in China, parents who violate it may lose their work and pay a fine to compensate the “cost of society”, and the Chinese government designed some policies to encourage family with only one child, such as more financial aid for medical problems, which may also contribute to our results [19]. Therefore, these findings need to be confirmed in future studies outside China.

In this study, family cohesion was found to be associated with behavioral problems among the adolescents with physically ill parents. These results were consistent with previous studies conducted in cancer and multiple sclerosis samples [23,24,25]. These results revealed that the quality of family environment, especially family cohesion, could help adolescents better adjust to parental physical illness psychologically, while the adolescents in less cohesive families were at higher risk of mental health problems. Collectivism focuses on community and stressed the importance of cohesion [27]. In China, family is considered as a community so that family members are prone to support each other, which may reduce behavioral problems of the adolescents with physically ill parents [28]. It may be a positive and feasible strategy to develop programs to increase family cohesion, thus improving adolescents’ mental health in the families with physically ill parents in the long run. For example, “Triple P-positive parenting program” has been shown to be effective in enhancing family relationships and attenuating adolescents’ psychological distress [42,43].

There are some limitations in this study. Firstly, we were unable to draw any causal conclusions because of the cross-sectional design, so all these findings need to be confirmed in future longitudinal studies. Secondly, this research was preliminary. Although we considered the effect of some characteristics of child and family in this study, future research could consider the effects of other aspects such as illness-related characteristics (e.g., illness duration and illness severity).Thirdly, the investigation was based on self-reported behavioral problems. Data obtained from parents and teachers should be considered in future study because data from multiple informants are more comprehensive.

## 5. Conclusions

To our knowledge, this is the first study focusing on the adolescents with physically ill parents in China. Our findings showed that Chinese adolescents with physically ill parents had more behavioral problems than those with healthy parents. In families with physically ill parents, the girls with siblings may have more behavioral problems than the girls without siblings; family cohesion was associated with behavioral problems. Hence, in the families with physically ill parents, interventions to enhance family cohesion may be positive and effective in reducing Chinese adolescents’ behavioral problems.

## Supplementary Files

Supplementary File 1
