# Supplementary material for: The Effects of Being an Only Child, Family Cohesion, and Family Conflict on Behavioral Problems among Adolescents with Physically Ill Parents
Source: Int J Environ Res Public Health. 2015 Sep 2;12(9):10910–22. doi: 10.3390/ijerph120910910 (PMC4586651; doi:10.3390/ijerph120910910)
Supplement: Supplementary File 1 [file ijerph-12-10910-s001.pdf]

*Supplementary Information*

# The Effects of Being an Only Child, Family Cohesion, and Family Conflict on Behavioral Problems among Adolescents with Physically Ill Parents

**Table S1.** Correlations between behavioral problems and adolescent's age, father's/mother's age, family cohesion and conflict.

| **Variable** | **Adolescents with Parental Physical Illness** | | | | | | | | | | |
|---|---|---|---|---|---|---|---|---|---|---|---|
| | **1** | **2** | **3** | **4** | **5** | **6** | **7** | **8** | **9** | **10** | **11** |
| 1.adolescent's age | 1 | **0.33** | **0.47** | −0.11 | 0.10 | **0.16** | **0.21** | 0.09 | 0.15 | 0.11 | 0.09 |
| 2.father's age | **0.29** | 1 | **0.65** | −0.02 | 0.02 | 0.07 | 0.06 | 0.07 | 0.07 | 0.13 | −0.03 |
| 3.mother's age | **0.38** | **0.77** | 1 | 0.10 | −0.04 | 0.01 | 0.03 | −0.03 | 0.05 | 0.05 | 0.04 |
| 4.family cohesion | −0.16 | **0.19** | 0.03 | 1 | **−0.55** | **−0.42** | **−0.37** | **−0.35** | **−0.28** | **−0.15** | 0.15 |
| 5.family conflict | 0.13 | −0.06 | 0.04 | **−0.66** | 1 | **0.28** | **0.33** | **0.25** | **0.18** | 0.08 | −0.11 |
| 6.behavioral problems | 0.14 | 0.03 | 0.04 | **−0.37** | **0.32** | 1 | **0.79** | **0.64** | **0.70** | **0.56** | **−0.19** |
| 7.emotional symptoms | 0.16 | −0.02 | 0.02 | **−0.33** | **0.25** | **0.85** | 1 | **0.37** | **0.49** | **0.33** | −0.08 |
| 8.conduct problems | 0.05 | 0.05 | 0.03 | **−0.28** | **0.19** | **0.77** | **0.54** | 1 | **0.32** | **0.25** | **−0.17** |
| 9.hyperactivity/inattention | **0.23** | 0.04 | 0.05 | **−0.35** | **0.30** | **0.78** | **0.53** | **0.50** | 1 | **0.17** | **−0.20** |
| 10.peer problems | −0.06 | 0.02 | 0.01 | −0.14 | **0.24** | **0.63** | **0.43** | **0.38** | **0.26** | 1 | **−0.21** |
| 11.prosocial behaviour | −0.02 | 0.05 | 0.02 | **0.29** | **−0.18** | **−0.31** | −0.16 | **−0.23** | **−0.31** | **−0.26** | 1 |
| **Variable** | **Adolescents without Parental Physical Illness** | | | | | | | | | | |
| | **1** | **2** | **3** | **4** | **5** | **6** | **7** | **8** | **9** | **10** | **11** |
| 1.adolescent's age | 1 | **0.39** | **0.45** | **−0.11** | **0.13** | **0.22** | **0.18** | **0.18** | **0.25** | 0.01 | −0.02 |
| 2.father's age | **0.37** | 1 | **0.78** | **−0.08** | **0.07** | **0.11** | **0.12** | **0.08** | **0.12** | **0.04** | −0.03 |
| 3.mother's age | **0.45** | **0.82** | 1 | **−0.09** | **0.10** | **0.11** | **0.13** | **0.07** | **0.12** | 0.02 | −0.01 |
| 4.family cohesion | **−0.10** | −0.02 | −0.01 | 1 | **−0.56** | **−0.39** | **−0.32** | **−0.32** | **−0.33** | **−0.21** | **0.27** |
| 5.family conflict | **0.11** | 0.03 | **0.05** | **−0.55** | 1 | **0.36** | **0.32** | **0.30** | **0.32** | **0.16** | **−0.20** |
| 6.behavioral problems | **0.17** | **0.09** | **0.09** | **−0.4** | **0.36** | 1 | **0.82** | **0.73** | **0.78** | **0.58** | **−0.33** |
| 7.emotional symptoms | **0.16** | **0.07** | **0.08** | **−0.31** | **0.31** | **0.84** | 1 | **0.47** | **0.54** | **0.36** | **−0.14** |
| 8.conduct problems | **0.18** | **0.10** | **0.09** | **−0.35** | **0.31** | **0.76** | **0.55** | 1 | **0.51** | **0.31** | **−0.29** |
| 9.hyperactivity/inattention | **0.15** | **0.09** | **0.08** | **−0.32** | **0.30** | **0.76** | **0.51** | **0.50** | 1 | **0.25** | **−0.31** |
| 10.peer problems | 0.01 | 0.02 | 0.01 | **−0.24** | **0.19** | **0.64** | **0.45** | **0.35** | **0.28** | 1 | **−0.30** |
| 11.prosocial behaviour | 0.02 | −0.02 | 0.02 | **0.34** | **−0.20** | **−0.35** | **−0.15** | **−0.30** | **−0.34** | **−0.30** | 1 |

Bold fonts: $p < 0.05$; Results for boys are below the diagonal and for girls are above the diagonal.

© 2015 by the authors; licensee MDPI, Basel, Switzerland. This article is an open access article distributed under the terms and conditions of the Creative Commons Attribution license (http://creativecommons.org/licenses/by/4.0/).
